# Regional Multiple Pathology Scores Are Associated with Cognitive Decline in Lewy Body Dementias

**DOI:** 10.1111/bpa.12182

**Published:** 2014-10-30

**Authors:** David R. Howlett, David Whitfield, Mary Johnson, Johannes Attems, John T. O'Brien, Dag Aarsland, Mitchell K.P. Lai, Jasinda H. Lee, Christopher Chen, Clive Ballard, Tibor Hortobágyi, Paul T. Francis

**Affiliations:** ^1^ Wolfson Centre for Age‐Related Diseases King's College London London UK; ^2^ Institute for Ageing and Health Newcastle University Newcastle upon Tyne UK; ^3^ Department of Psychiatry University of Cambridge Cambridge UK; ^4^ Department of Neurobiology, Ward Sciences and Society Karolinska Institute Stockholm Sweden; ^5^ Centre for Age‐Related Medicine Stavanger University Hospital Stavanger Norway; ^6^ Department of Pharmacology Yong Loo Lin School of Medicine National University of Singapore Singapore Singapore; ^7^ Department of Neuropathology Institute of Pathology University of Debrecen Debrecen Hungary

**Keywords:** Alzheimer's disease, cognitive decline, dementia with Lewy bodies, Lewy body dementia, Parkinson's disease dementia

## Abstract

Dementia with Lewy bodies (DLB) and Parkinson's disease dementia (PDD) are characterized by the presence of α‐synuclein‐containing Lewy bodies and Lewy neurites. However, both dementias also show variable degrees of Alzheimer's disease (AD) pathology (senile plaques and neurofibrillary tangles), particularly in areas of the cortex associated with higher cognitive functions. This study investigates the contribution of the individual and combined pathologies in determining the rate of cognitive decline. Cortical α‐synuclein, phosphorylated tau (phosphotau) and Aβ plaque pathology in 34 PDD and 55 DLB patients was assessed semi‐quantitatively in four regions of the neocortex. The decline in cognition, assessed by Mini Mental State Examination, correlated positively with the cortical α‐synuclein load. Patients also had varying degrees of senile Aβ plaque and phosphotau pathology. Regression analyses pointed to a combined pathology (Aβ plaque plus phosphotau plus α‐synuclein‐positive features), particularly in the prefrontal cortex (BA9) and temporal lobe neocortex with the superior and middle temporal gyrus (BA21, 22), being a major determining factor in the development of dementia. Thus, cognitive decline in Lewy body dementias is not a consequence of α‐synuclein‐induced neurodegeneration alone but senile plaque and phosphorylated tau pathology also contribute to the overall deficits.

## Introduction

The pathological substrate of dementia and cognitive decline in Lewy body dementias is an important issue for the development of biomarkers to measure outcome and for intervention studies. In both dementia with Lewy bodies (DLB) and Parkinson's disease dementia (PDD), the severity of dementia is often considered to be a function of cortical Lewy body formation 12 although senile plaques and neurofibrillary tangles, the pathognomic features of Alzheimer's disease (AD), are also found in DLB and PDD 14, 21. There has been considerable recent interest in this area with a number of studies focusing on the overlap of Alzheimer and Lewy body pathologies, although few reports have included cognitive or other clinical assessments of severity. Two recent studies 11, 24, despite only including patients with PDD and PD (and not DLB), are perhaps the most informative, highlighting the possible importance of cortical Aβ and phosphorylated tau (phosphotau) in the rate of decline into dementia. Analyses in other studies, however, support the role of α‐synuclein in determining the decline in cognitive state in both PDD and DLB 23, 43.

To address this issue, in our study, neuropathology (senile plaques, phosphotau pathology and α‐synuclein‐positive Lewy bodies and neurites) has been analyzed in groups of DLB and PDD patients where we were able to assess this pathology with respect to rate of cognitive decline determined by serial assessments over the years of dementia 1. This novel approach, therefore, allows the provision of significant support for the hypothesis that all three cortical pathologies relate to the premortem rate of cognitive decline to establish that all three pathologies play a role in the development of dementia.

## Materials and Methods

### Assessment of cases

All cases were selected based on clinicopathological consensus diagnoses. Clinical classification of PDD was made when parkinsonism preceded dementia by more than a year 34; diagnosis of DLB was made when cognitive impairment or hallucinations were present before or within 1 year of onset of parkinsonism. All cases were prospectively assessed by experienced clinicians using validated clinical rating instruments. AD cases were clinically diagnosed on the basis of meeting the Consortium to Establish a Registry for Alzheimer's Disease (CERAD) criteria for a diagnosis of probable or definite AD, DLB according to international consensus criteria 34 and PDD according to Movement Disorders Society criteria 16. Neuropathological assessment was performed according to standardized neuropathological scoring/grading systems, including neurofibrillary Braak staging (threads and tangles), CERAD scores (neuritic plaques), Newcastle/McKeith Criteria for Lewy body disease (Lewy bodies and neurites), National Institute on Aging—Alzheimer's Association guidelines and phases of amyloid‐β (Aβ) deposition 5, 34, 37, 45. Cognitive impairment data were assessed annually and are presented as the annual decline in Mini Mental State Examination (MMSE) scores from the time of dementia diagnosis, usually over the 8–10 years prior to death (MMSE decline). The demographics and clinical state of the PDD, DLB and AD patients is shown in Table 1. Full details of the patient cohorts are presented in Supplementary Table S1. More details of the selection and clinical assessment procedures can be seen elsewhere (1—Newcastle and Oxford cohorts).

**Table 1:** Demographics and clinical data of patients included in the study

	n	Age (years)	Gender M/F	PMD (h)	Braak	Years of dementia	MMSE last	MMSE decline per year
PDD	34	79.9 ± 6.0	12/22	33.5 ± 15.6	0–5	4.1 ± 2.7	12.7 ± 8.0	2.0 ± 1.6
DLB	55	81.7 ± 6.5	39/16	41.3 ± 28.0	1–6	6.2 ± 3.1	14.1 ± 8.1	3.6 ± 3.6
PDD + DLB	89	80.2 ± 6.4	51/38	37.3 ± 24.2	0–6	5.3 ± 3.1	13.4 ± 8.0	2.9 ± 2.9
AD	16	88.1 ± 7.8	5/11	25.2 ± 21.6	4–6	10.1 ± 2.6	8.6 ± 7.6	3.8 ± 3.7
Control	25	79.7 ± 7.6	14/11	35.4 ± 22.3	0–2			

“Braak” refers to NFT Braak stage. “MMSE last” is the score at the last interview before death. “MMSE decline” is the decline per year averaged over the period of clinical observation and was usually 8–10 years. No MMSE data are available in the aged control group. Data are expressed as mean ± standard deviation.

Abbreviations: AD = Alzheimer's disease; DLB = dementia with Lewy bodies; MMSE = Mini Mental State Examination; PDD = Parkinson's disease dementia; PMD = post mortem delay.

### Brain tissue

Tissue was provided (seven micron wax sections) by the University Hospital Stavanger (27 cases), Newcastle University (21 cases) and the Thomas Willis Oxford (17 cases) Brain Collections and the London Neurodegenerative Diseases Brain Bank (65 cases), the UK sites being part of the Brains for Dementia Research network. In all cases, formalin fixation time was less than 3 months. Samples from prefrontal cortex (BA9), temporal lobe neocortex with the superior and middle temporal gyrus (BA21, 22), anterior cingulate cortex (BA24) and inferior parietal lobe neocortex (BA40) were studied. In total, 55 DLB patients (age 65–92), 34 PDD patients (age 68–89), 16 AD patients (age 72–103 years) and 25 aged controls (age 65–96) were included. All participants gave informed consent for their tissue to be used in research and the study had ethical approval (08/H1010/4). Control cases (from the above brain banks) were neurologically normal, with only mild, age‐associated neuropathological changes and no history of psychiatric disease. The pathological features in these latter subjects were not of sufficient severity to change the classification of them from being neurologically normal, aged controls.

### Immunohistochemistry

Labeling of neuropathology was undertaken by standard protocols. Briefly, sections were dewaxed and rehydrated through xylene and descending concentrations of alcohol into water. For Aβ and phosphotau, antigen retrieval was carried out by either immersion in 98% formic acid for 6 h (for Αβ labeling) or microwaving for 10 minutes in citrate buffer pH 6.0 (phosphotau). For α‐synuclein, antigen retrieval was carried out by autoclaving for 10 minutes in ethylenediaminetetraacetic acid buffer pH 8.0 followed by immersion for 15 minutes in 98% formic acid. Endogenous peroxidases were subsequently blocked by 0.3% hydrogen peroxide in Tris‐buffered saline (30 minutes). Primary antibodies were added (Αβ, 1:1000 Dako M0872, Dako Ltd., Ely, Cambs., UK; phosphotau, AT8 1:4000 Thermo Scientific MN1020, Thermo Fisher Scientific, Cramlington, Northumberland, UK; α‐synuclein, NCL‐SYN 1:30 Novacastra Laboratories, Newcastle upon Tyne, UK) and tissue sections incubated 1 h at room temperature. Subsequent horseradish peroxidase (HRP) and diaminobenzidine color development was performed using Menarini X‐Cell plus polymer HRP detection kit (MP‐XCPDAD‐U100, Menarini Diagnostics, Winnersh‐Wokingham, Berks., UK).

### Semi‐quantitative pathology scoring

Senile plaques were assessed by Aβ labeling. Neurofibrillary tangles, neuritic plaques, dystrophic neurites and neuropil threads were detected and evaluated by a combination of phosphotau immunohistochemistry (AT8 antibody) and silver impregnation (Gallyas or modified Bielschowsky) 2. Assessment of α‐synuclein pathology included Lewy neurites and Lewy bodies. A semi‐quantitative assessment was preferred to a morphometric approach in order to provide a means for considering summated pathologies. Semi‐quantitative assessments of senile Aβ plaques, phosphotau and α‐synuclein pathology were conducted blind to clinical diagnosis, by experienced neuropathologists, using a four‐tiered scale of 0 (none), 1 (sparse), 2 (moderate) and 3 (severe/frequent) to score sections from each brain area, as described previously 48. All cases were reviewed by a single neuropathologist to confirm that there was no interobserver variability. After preliminary observations, it was reasoned that the low AD group number and severe plaque and phosphotau pathology would skew the overall statistical comparisons; the AD patients were subsequently excluded from statistical analyses principally aimed at exploring pathology and cognition in the Lewy body diseases, although the pathology data are included in order to provide a benchmark for the level of plaque and phosphotau occurrence in DLB, PDD and controls, compared with AD.

### Immunoassays

Briefly, samples were treated with 8 × 5 M guanidine, 50 mM Tris‐HCl buffer at room temperature for 4 h, followed by dilution with 1:50 cold reaction buffer [50 mM Tris‐HCl buffer, pH 7.5 with addition of protease inhibitors (Roche Diagnostics, Burgess Hill, West Sussex, UK) and 2 μg/mL pepstatin A (Sigma Aldrich, Gillingham, Dorset, UK)]. Guanidine/Tris‐HCl homogenates were assayed in duplicate as recommended by the manufacturer for the Aβ_42_ enzyme‐linked immunosorbent assay (ELISA) kit (#KHB3442), Aβ_40_ ELISA kit (#KHB3482), tau pS396 ELISA kit (#KHB7031) and total tau ELISA kit (#KHB0042) (all InVitrogen, Life Technologies, Paisley, Scotland).

### Statistical analysis

Non‐parametric bivariate correlations and regression analyses (stepwise method in SPSS, IBM UK Ltd., Portsmouth, UK) were used to determine relationships between variables. Correlations were determined considering MMSE decline per year and regional pathologies as variables. Preliminary analyses (not shown) considered the MMSE score at the last interview before death; the correlations generated suggested that this factor was not as important as MMSE decline as a dependent variable. Backward regression analyses employed MMSE decline per year as the dependent variable and the brain area pathologies as independent variables. Where stated, groups were compared by Student's *t*‐test.

## Results

**Figure 1:**
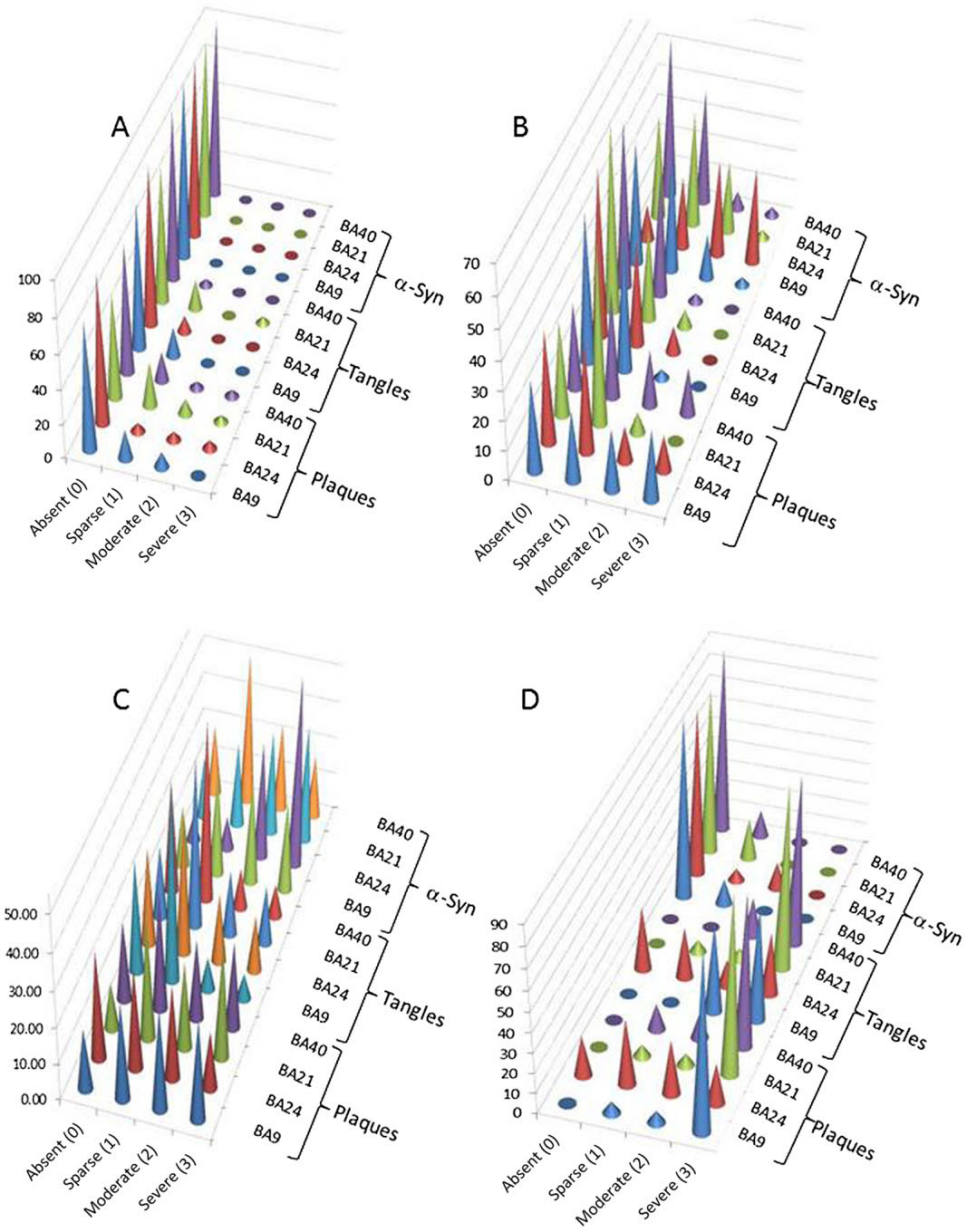
Frequency of pathology scores in (**A**) control, (**B**) Parkinson's disease dementia, (**C**) dementia with Lewy bodies and (**D**) Alzheimer's disease. The frequency of each pathology score, by brain region, was calculated as described in “Materials and Methods” section. The left‐hand *y*‐axis represents the percentage of cases with a particular score. BA9, BA21, BA24 and BA40 refer to the Brodmann areas, as defined in Materials and Methods section. “α‐Syn” is α‐synuclein labeling of Lewy bodies and neurites; “tangles” refer to phosphorylated tau labeling of neurofibrillary tangle and neurites; “plaques” is labeling of senile plaques with an antibody to Aβ.

The semi‐quantitative scores for frequency of senile Aβ plaques (0–3), phosphotau (0–3) and α‐synuclein pathology (0–3) in the three disease states and healthy controls are shown in Figure 1. The control cases showed varying frequencies of Αβ‐positive senile plaques in all four cortical regions studied, a scarcity of neurofibrillary pathology and an absence of α‐synuclein pathology. Overall, PDD was characterized by α‐synuclein inclusions and senile Αβ‐positive plaques in the four regions of cortex, with a lesser incidence of neurofibrillary tangles (NFT's). DLB patients, however, exhibited marked plaque, phosphotau and α‐synuclein pathology throughout the cortical regions. The AD patients, included for comparative purposes, showed severe plaque and phosphotau pathology in prefrontal cortex (BA9), temporal lobe neocortex with the superior and middle temporal gyrus (BA21, 22), and inferior parietal lobe neocortex (BA40), somewhat less in anterior cingulate cortex (BA24) and a fairly sparse occurrence of Lewy bodies (Figure 1 and Table 2).

**Table 2:** Individual pathology scores for senile plaques, neurofibrillary tangles and α‐synuclein inclusions in four cortical areas in PDD, DLB, AD and control cases

	Plaque BA9	Plaque BA21	Plaque BA24	Plaque BA40
Control	0.35 ± 0.65	0.57 ± 0.84	0.29 ± 0.78	0.39 ± 0.78
PDD	1.39 ± 1.10	0.74 ± 0.88	0.97 ± 0.83	1.23 ± 0.91
DLB	1.68 ± 1.07	1.73 ± 1.04	1.28 ± 1.07	1.47 ± 1.09
AD	2.81 ± 0.54	2.81 ± 0.54	1.47 ± 1.06	2.63 ± 0.72
	**Tangles BA9**	**Tangles BA21**	**Tangles BA24**	**Tangles BA40**
Control	0.17 ± 0.39	0.30 ± 0.70	0.10 ± 0.30	0.04 ± 0.21
PDD	0.53 ± 0.61	0.44 ± 0.59	0.53 ± 0.63	0.48 ± 0.72
DLB	0.93 ± 0.84	1.31 ± 0.98	1.23 ± 1.02	0.98 ± 0.89
AD	2.56 ± 0.51	2.81 ± 0.54	1.44 ± 1.26	2.81 ± 0.40
	**α‐Synuclein BA9**	**α‐Synuclein BA21**	**α‐Synuclein BA24**	**α‐Synuclein BA40**
Control	0.00	0.00	0.00	0.00
PDD	0.79 ± 1.06	0.94 ± 0.81	1.85 ± 1.04	0.59 ± 1.77
DLB	1.62 ± 1.05	1.75 ± 1.09	2.28 ± 0.92	1.39 ± 0.98
AD	0.13 ± 0.34	0.20 ± 0.41	0.31 ± 0.70	0.13 ± 0.34

Areas studied were BA9 (prefrontal cortex), BA21, 22 (temporal lobe neocortex with the superior and middle temporal gyrus), BA24 (anterior cingulate cortex) and BA40 (inferior parietal lobe neocortex). Plaque, tangle and α‐synuclein pathology was assessed on a semi‐quantitative scale as described in Materials and Methods section. Data are means ± standard deviations from the scoring of patients shown in Table 1.

Abbreviations: AD = Alzheimer's disease; DLB = dementia with Lewy bodies; PDD = Parkinson's disease dementia.

The possibility of MMSE decline being a product of a summation of the various pathologies was considered in the individual patient groups. The semi‐quantitative scores (0–3) for plaques, phosphotau and α‐synuclein pathology were summed to give an overall pathology measure for each region (0–9). In these groups, the pathology summation score (PSS) correlated with the MMSE decline for the PDD and PDD + DLB patients in BA9, BA21 and BA40 (Table 3A). Only in BA21 was there a correlation between PSS and MMSE decline in the DLB group. The major contributor to these correlations would appear to be the α‐synuclein component as a number of significant correlations were also observed between α‐synuclein scores and MMSE decline in PDD and PDD + DLB groups (and BA21 in the DLB patients) (Table 3A). It was noticeable, however, that the *R* values for PSS vs. MMSE decline in the PDD group was greater than that for α‐synuclein alone vs. MMSE decline in all four brain areas studied, suggesting a possible summation effect. There were, additionally, significant correlations between cortical plaque and MMSE decline in the PDD group and cortical phosphotau and MMSE decline in the PDD + DLB combined group (Table 3A), although there were no differences in rate of MMSE decline between the NFT Braak stages (data not shown). There were no significant associations between cognitive decline and age or gender (data not shown).

**Table 3:** Correlations between MMSE decline and pathology scores in PDD and DLB patients in BA9 (prefrontal cortex), BA21, 22 (temporal lobe neocortex with the superior and middle temporal gyrus), BA24 (anterior cingulate cortex) and BA40 (inferior parietal lobe neocortex)

(A)		n	PSS	α‐Synuclein	Plaques	Tangles
BA9	PDD + DLB	65–68	***0.343***	***0.45***	0.053	***0.264***
	PDD	30–32	***0.509***	***0.501***	0.303	0.291
	DLB	35–37	0.176	***0.333***	−0.139	0.183
BA21	PDD + DLB	65–69	***0.406***	***0.467***	0.231	***0.291***
	PDD	28–32	***0.358***	0.156	0.359	0.302
	DLB	36–37	***0.423***	***0.577***	0.127	0.217
BA24	PDD + DLB	64–68	0.227	***0.315***	0.142	0.139
	PDD	32	0.255	0.109	0.255	0.268
	DLB	32–36	0.132	0.258	0.129	0.036
BA40	PDD + DLB	65–69	***0.389***	***0.475***	0.067	***0.283***
	PDD	29–32	***0.441***	0.309	***0.370***	0.186
	DLB	36–37	0.273	***0.474***	−0.105	0.272
**(B)**		**n**	**Αβ42**	**Αβ40**	**Ratio Aβ42:Aβ40**	
BA9	PDD + DLB	69	***0.385***	***0.314***	***0.340***	
	PDD	32	***0.407***	***0.345***	***0.45***	
	DLB	37	***0.373***	0.21	0.24	
BA21	PDD + DLB	68	***0.409***	0.088	0.240	
	PDD	31	***0.450***	0.126	0.290	
	DLB	37	0.307	0.099	0.09	

(A) Correlation (*R*s) of pathology summary scores and individual pathology scores with MMSE decline per year. PSS is the summation of α‐synuclein, plaque and tangle scores.

(B) Correlation (*R*s) of Aβ40 and Aβ42 enzyme‐linked immunosorbent assay data with MMSE decline per year.

Values are *R*s and those indicated in ***large bold italics*** indicate statistically significant correlations (*P* < 0.05) between that variable and MMSE decline.

Abbreviations: DLB = dementia with Lewy bodies; MMSE = Mini Mental State Examination; PDD = Parkinson's disease dementia; PSS = pathology summation score.

Guanidine HCl extracts of BA9 and BA21 tissue were analyzed by ELISA for Αβ40, Αβ42, total tau and phosphorylated serine 396 tau. Both BA9 and BA21 Αβ40 and Αβ42 concentrations correlated significantly with the respective plaque scores for the two areas (*P* < 0.001 for all comparisons) and BA9 and BA21 phosphorylated serine 396 tau (but not total tau) correlated with NFT scores (*P* < 0.0001) (data not shown). MMSE decline for the PDD + DLB and PDD alone groups was found to correlate significantly with BA9 Αβ40 and Αβ42 concentrations (Table 3B). BA9 Αβ40 and Αβ42 to Αβ40 ratio also correlated with MMSE decline in these patient groups as did BA21 Αβ42. In the DLB patients, only Αβ42 demonstrated a significant correlation with MMSE decline. There were no significant correlations with MMSE decline when total tau or phosphorylated serine 396 tau data were assessed (data not shown).

A series of backward stepwise regression analyses were conducted to assess the major pathological variable(s) with respect to MMSE decline. For the PDD + DLB and DLB only groups, this variable was BA21 α‐synuclein pathology while for the PDD group it was BA9 α‐synuclein (Table 4). Based on the observed significant correlations between MMSE decline and both PSS and α‐synuclein in BA9, BA21 and BA40 (Table 3A), a similar backward stepwise regression analysis was conducted. Considering the individual pathologies, BA21 α‐synuclein was the major significant variable in the DLB and grouped PDD + DLB patients and BA9 α‐synuclein in PDD patients. When a regression analysis of α‐synuclein and PSS was undertaken, BA21 PSS was the greater determinant in DLB and PDD + DLB (Table 4).

**Table 4:** Regression analysis of α‐synuclein, Aβ and phosphorylated tau pathology in BA9, BA21, BA24 and BA40 in PDD and DLB patient groups

(A) All pathologies				
		Beta	t	Sig
PDD + DLB	BA21 α‐synuclein	0.417	3.381	0.001
PDD	BA9 α‐synuclein	0.372	2.043	0.053
DLB	BA21 α‐synuclein	0.603	3.929	0.001
**(B) PSS and α‐synuclein**				
		**Beta**	**t**	**Sig**
PDD + DLB	BA21 PSS	0.767	4.565	<0.0001
PDD	BA9 PSS	0.566	3.434	0.002
DLB	BA21 PSS	0.937	4.575	<0.0001
	BA9 PSS	−0.586	−2.861	0.008

(A) All three individual pathologies or (B) α‐synuclein and PSS only were analyzed in a backward stepwise regression model in BA9 (prefrontal cortex), BA21, 22 (temporal lobe neocortex with the superior and middle temporal gyrus), BA24 (anterior cingulate cortex) and BA40 (inferior parietal lobe neocortex).

Abbreviations: DLB = dementia with Lewy bodies; PDD = Parkinson's disease dementia; PSS = pathology summation score.

## Discussion

This study investigates the role of individual and combined pathologies in the rate of cognitive decline in clinically and pathologically defined Lewy body dementia cases. The data indicate that although there were significant correlations between Lewy body incidence and cognitive decline across a number of cortical regions, a pathological assessment, where scores for senile Aβ plaque, phosphotau and α‐synuclein loads were combined, provided an excellent correlate in both PDD and DLB patients.

The rate of decline in cognition is a more appropriate clinical measure than a single determination at the last interview before death 1. Our analyses, therefore, link the development of dementia in the years before death with the incidence of the different neuropathologies. Agreement on the involvement of AD‐type pathologies in the Lewy body dementias is not universal and may reflect the differing means of assessing dementia. In a group of PDD patients, cortical plaque (but not α‐synuclein) and MMSE score at the last interview before death was predictive of a shorter time to dementia and a combination of the pathologies was found to be a greater predictor of dementia than the individual loads 11. Cortical plaque was also associated with a shorter survival time in PDD patients 28. A study of over 100 PDD patients reported that Lewy body score, Braak neurofibrillary tangles and plaque densities were greater than in a group of non‐demented PD patients and the plaque and tau loads correlated with the state of dementia 23. The degree of dementia in a large DLB cohort (>150 patients), determined longitudinally by a battery of cognitive function tests, was found to be associated with Lewy body pathology and was independent of amyloid and tau pathology 43, while in a small (22 patients) group of DLB patients cortical and subcortical Aβ pathology correlated with the incidence of Lewy bodies, although no clinical measures were described 10.

Classically, AD patients have been defined by the presence of senile plaques and NFTs, and Lewy body dementias by α‐synuclein‐positive intracellular inclusions and neurites. Increasingly, however, data point to a “graying” of the pathological boundaries between these disorders and the occurrence of multiple neuropathologies, the “multimorbidity” theory 3. Equally, other studies have suggested that AD pathology in particular plays a role in cognitive decline in Lewy body dementias 11, 20, 23, 36. In agreement with previous data 28, the present study illustrates that it is not uncommon to observe α‐synuclein, senile Αβ plaque and phosphotau pathology in all three dementias. Studies have previously attempted to relate the cognitive decline in PDD and DLB to the presence of AD pathology with conflicting results, the latter probably arising as a consequence of issues relating to diagnosis and sample population 29. We attempted a relatively crude assessment of the total pathology in any brain region studied by simply adding together the individual pathology scores for α‐synuclein, Αβ‐positive plaques and phosphotau to give a PSS. The analysis showed that despite the importance of α‐synuclein pathology, particularly in BA9 and BA21, the combined pathology score for plaques, phosphotau and α‐synuclein‐positive features (the PSS) in these areas was a major variable determining the severity of the cognitive deficit, as defined by MMSE decline, in the individual PDD and DLB patient groups. Although MMSE scoring was developed primarily for assessing cognitive decline in AD, our data do highlight its usefulness in other dementias. The implication of the involvement of BA9 (part of the dorsolateral prefrontal cortex) is supportive of Lewy body dementia patients showing deficits in executive function 33, which is thought to be largely subserved through the dorsolateral prefrontal cortex 8. Similarly, the temporal cortex (which includes BA21) is believed to be involved in memory and visuospatial recognition, key features in PDD and DLB. Thus, the occurrence of BA9 and BA21 Lewy pathology can be associated with the deficiencies in neurological functions characteristic of PDD and DLB patients. Neurofibrillary tangle density and α‐synuclein load have also been reported to be instrumental in the cognitive decline over 13 years in a cohort of 354 Catholic nuns, priests and brothers 49, suggesting a multipathology role in cognitive decline in old age.

The two forms of dementia associated with Lewy pathology, DLB and PDD are usually distinguished and defined clinically by the temporal relationship between the respective onsets of dementia and motor defects 11, and commonality exists for Lewy body presence and distribution associated with the two dementias with little difference in neuropathology 13. An association of plaque pathology with the rate of cognitive decline in DLB patients and a greater plaque burden in DLB than PDD has been reported 18, 42. Despite also observing a greater plaque (and tangle) load in the present group of DLB patients, there was no association between MMSE decline and plaque or phosphotau pathology. Only by grouping all Lewy body patients together (PDD + DLB) and ignoring the clinical division between PDD and DLB patients was there a correlation between the rate of MMSE decline and the incidence of phosphotau pathology (BA9, BA21 and BA40), although these correlations were never as strong as between PSS and rate of MMSE decline.

Employing Lewy body dementia groups with >30 patients, we observed that the decline in cognition in the years before death in the DLB patients correlated with cortical Lewy pathology (defined by α‐synuclein immunohistochemistry) in BA9, BA21 and BA40; in the PDD patients a correlation with only BA9 α‐synuclein immunohistochemistry was observed. The role of cortical α‐synuclein and plaque pathologies in cognitive decline in PDD is supported by data showing that the duration of parkinsonian symptoms prior to dementia is associated with less severe cortical α‐synuclein and lower plaque scores 4 and cerebral plaque deposition (assessed by PiB binding) was lower in PD patients at risk of dementia than in cognitively normal control subjects 40. However, the presence of fibrillar Aβ, as detected by PET scanning, has been described in PD patients with early dementia 6, 7. In other words, it is during phases of cognitive decline that these pathologies play a major role.

The relevance of plaque Αβ to neurodegeneration and cognitive decline has been widely debated 13, 15, 39, with the suggestion that soluble oligomeric forms of the protein play a greater role in neuronal loss 44, 46, 47. In the present study, guanidine extractable Αβ40 and Αβ42 was found to correlate with plaque Αβ, and in BA9, these Αβ species correlated with MMSE decline in both the PDD and combined PDD + DLB patient groups. Although the soluble oligomeric component of these extracts may play a role in cognitive decline, the fact that the proportion of extractable (SDS, guanidine hydrochloride, formic acid) to freely soluble Αβ is extremely high 27, 41 suggests that the less soluble forms requiring dissolution in guanidine hydrochloride are likely to be of significance in the dementia process.

There is clearly a mixed morbidity state in these dementias although the etiology is not clear. In both familial AD (FAD) and Down syndrome (DS), classical AD pathology can be accompanied by the presence of Lewy bodies and Lewy neurites 30, 31. As well as forming the cornerstone of the “amyloid hypothesis” of AD 22 in demonstrating that mutations in the APP gene or its overexpression, directly or indirectly, both drive the formation of NFTs, these studies in FAD and DS patients also point to a link between amyloidogenesis and α‐synuclein aggregation 35. Proteins that are susceptible to mis‐folding are also likely to promote the aggregation of each other. For example, Αβ and α‐synuclein are able to act as seeds and affect aggregation of each other 32 and tau and α‐synuclein can interact and promote synergistic fibrillization 17, 19. The biophysical features of AD pathology (oligomeric/fibrillar Aβ and aggregated phosphotau) may act synergistically with α‐synuclein to promote the aggregation and spread of α‐synuclein pathology 24. The seminal seeds of dementia, therefore, while providing the definition of the disease as AD or PDD/DLB, may be sufficient to facilitate the development of the alternate pathologies that we and others have observed. Certainly in a transgenic mouse model of AD, the introduction of a human mutant α‐synuclein transgene promoted both the Αβ and tau pathology and also accelerated the cognitive decline, pointing to a synergistic effect 9.

Although we studied the main neurodegenerative pathologies in areas of the neocortex associated with higher cognitive functions, a limitation of the study is that non‐cortical brain regions with dementia‐linked pathology, such as the striatum 26 or thalamus 25, were not included in this study. Furthermore, other potentially relevant pathologies were not assessed, including vascular pathology and TDP‐43. Such features may be of relevance although assessment criteria for vascular pathologies await standardization 2 and TDP‐43 pathology is less frequent in pure synucleinopathies than in diseases where tauopathy is the major pathology 38. A strength of the present study is the prospective design of the cohorts that allowed the calculation of the annual rate of cognitive decline prior to death. This study, while not providing unequivocal evidence that the dementia observed in Lewy body diseases is a function of a multipathological insult by senile Αβ plaque, phosphotau and α‐synuclein, does point to the multiple pathologies as playing a role in cognitive decline in these diseases, findings that may have important implications in terms of clinical practice and research.

Undoubtedly, the presence of α‐synuclein‐positive pathology is a major correlate of cognitive decline in PDD and DLB patients. These patients, however, develop variable levels of all three pathologies and, in many cases, the summated deposition of these proteins, as assessed by semi‐quantitative scoring, correlated with the decline in MMSE score. This relationship was the most striking in BA9 and BA21, areas of major significance in AD but which also appear to be determinants of cognitive decline in PDD and DLB. Thus, using a rather simple and easily reproducible means of assessment based on standard and routinely applied neuropathological criteria, semi‐quantitative scoring has provided evidence that dementia in PDD and DLB is governed by multiple pathological features in addition to their characteristic Lewy bodies.

## Conclusions

Pathological assessment of Lewy body dementia and AD patient groups demonstrated Αβ‐positive senile plaques plus phosphotau‐ and α‐synuclein‐positive pathologies. The presence of any of the pathologies could not be considered a defining feature of any particular dementia as evidence for each was observed across the entire patient group. Obviously, attempting to correlate end‐stage pathology to cognitive decline in the latter years of life of any dementia patient, as in the present study, may be open to criticism. Nevertheless, summated pathology scores, particularly in areas BA9 and BA21 in the PDD and DLB patients, demonstrated a significant relationship with the decline in MMSE scores averaged over the years before death, supporting the view that the summated score was a better predictor of decline.

## Conflict of Interest

The authors have no conflicts of interest to declare.

## Supporting information


**Table S1.** Summary of cases included in the study. “nd” denotes no data available. No MMSE scoring was recorded for any of the control cases.
