# Supplementary material for: Regional Multiple Pathology Scores Are Associated with Cognitive Decline in Lewy Body Dementias
Source: Brain Pathol. 2014 Oct 30;25(4):401–8. doi: 10.1111/bpa.12182 (PMC8029273; doi:10.1111/bpa.12182)
Supplement: Supplementary file 1 — Table S1. Summary of cases included in the study. “nd” denotes no data available. No MMSE scoring was recorded for any of the control cases. [file BPA-25-401-s001.rtf]

		Gender	Age at death	Year of death	Post
mortem delay
(hr)	NIA-AA Level of AD Neuropathological Change	Braak stage	MMSE score at last interview before death	MMSE average decline/
year	
Control	1	F	82	2006	43	Low	nd			
cases	2	F	87	2002	21	0	0			
	3	M	81	2009	42	Low	1			
	4	M	79	2003	34	Low	nd			
	5	F	90	2010	74	Low	1/2			
	6	M	85	1995	48	nd	nd			
	7	M	86	2000	6	Low	2			
	8	F	89	2010	65	Low	2			
	9	M	71	2001	5	0	0			
	10	F	68	2000	9	0	0			
	11	M	80	2004	48	Intermediate	2			
	12	F	76	1997	63	0	0			
	13	M	80	1996	11	0	0			
	14	F	79	1995	38	Intermediate	2			
	15	M	77	1996	29	Low	1			
	16	M	66	2009	52	Low	nd			
	17	M	70	1996	45	0	0			
	18	M	80	1995	35	Low	1			
	19	M	77	1994	96	nd	nd			
	20	F	96	1996	72	Low	2			
	21	M	85	1995	16	Intermediate	2			
	22	F	80	2008	22	Low	nd			
	23	M	85	1997	42	Intermediate	3			
	24	M	65	1996	29	0	0			
	25	F	80	1995	31	Low	1			
										
PDD	26	M	70	2002	17	Low	2	16	1.8	
patients	27	F	69	2003	46	Low	2	19	0.0	
	28	F	73	2003	30	Intermediate	4	5	5.5	
	29	M	81	2003	40	Low	2	6	1.9	
	30	M	75	2003	40	Intermediate	3	17	5.0	
	31	M	79	2004	30	Intermediate	3	0	2.2	
	32	M	76	2004	17	0	2	0	2.3	
	33	M	68	2004	11	Severe	5	9	2.2	
	34	M	73	2005	31	0	0	19	0.0	
	35	M	89	2005	64	Intermediate	3	4	6.3	
	36	F	89	2000	54	Low	2	0	0.0	
	37	F	83	2001	24	Low	1/2	12	2.1	
	38	M	83	2001	37	Low	1/2	16	0.5	
	39	M	75	2001	36	Low	1/2	16	1.5	
	40	F	85	2001	nd	Intermediate	3/4	2	3.4	
	41	M	79	2002	72	Severe	5/6	9	2.5	
	42	M	82	2002	24	Low	1/2	20	1.0	
	43	F	73	2002	60	Low	2	25	0.3	
	44	F	80	2002	28	Low	1/2	29	0.0	
	45	F	81	2002	28	Low	2	26	0.2	
	46	M	78	2002	24	Intermediate	3	5	2.4	
	47	F	88	2002	72	Low	2	13	3.8	
	48	M	80	2002	26	Low	2	20	1.1	
	49	M	72	2002	9	Low	1	27	-0.6	
	50	M	79	2002	30	Low	1/2	10	1.8	
	51	F	84	2002	27	Low	1/2	0	0.0	
	52	F	85	2002	36	Low	1/2	3	3.3	
	53	F	83	2003	24	Low	1/2	14	2.8	
	54	F	75	2003	24	Low	1/2	12	2.0	
	55	F	82	2003	33	Low	1/2	6	2.4	
	56	M	88	2003	24	Intermediate	3/4	15	3.3	
	57	F	86	2004	24	Low	2	11	2.3	
	58	F	88	2004	32	Intermediate	3/4	3	2.3	
	59	M	86	2004	32	Low	0	16	1.4	

Supplementary Table 1.  Summary of cases included in the study.  'nd' denotes no data available.  No MMSE scoring was recorded for any of the control cases.

										
DLB	60	F	87	1993	13	Severe	5/6	11	3.7	
patients	61	M	85	1993	19	Severe	5/6	0	1.0	
	62	M	82	1991	80	Severe	5/6	6	4.0	
	63	M	82	1994	29	Intermediate	3/4	9	2.7	
	64	M	81	1998	38	Intermediate	3/4	27	0.0	
	65	F	88	1999	34	Intermediate	3/4	30	0.0	
	66	F	88	2003	16	Intermediate	3	18	1.5	
	67	M	77	2003	65	Intermediate	3	12	4.0	
	68	F	75	2004	64	Severe	6	6	4.0	
	69	M	77	2004	29	Low	2	nd	6.0	
	70	F	91	2005	84	Severe	5	nd	1.0	
	71	F	75	2005	78	Severe	6	15	4.0	
	72	M	76	2006	13	Low	2	nd	5.0	
	73	M	74	2007	42	Intermediate	4	12	3.5	
	74	M	71	2007	8	Low	2	1	3.7	
	75	F	80	2008	17	Severe	5	nd	nd	
	76	M	77	2010	46	Intermediate	3	12	2.2	
	77	F	87	2008	24	Severe	5/6	17	0.4	
	78	F	92	2008	96	Intermediate	3/4	10	1.8	
	79	F	92	1999	60	Low	1/2	14	-1.0	
	80	M	76	2003	70	Low	1/2	16	2.2	
	81	M	75	2000	76	Intermediate	3/4	15	5.3	
	82	M	84	2001	74	Intermediate	3/4	18	3.3	
	83	F	85	2000	38	Intermediate	3/4	7	7.5	
	84	M	86	2001	115	Intermediate	3/4	7	0.0	
	85	M	77	2002	57	Intermediate	5/6	16	7.0	
	86	F	76	2008	96	Low	1/2	14	1.0	
	87	M	74	2007	20	Severe	5/6	24	0.5	
	88	M	81	2010	85	Intermediate	3/4	10	6.7	
	89	F	83	2008	14	Intermediate	3/4	0	0.0	
	90	F	87	2010	33	Low	1/2	0	0.0	
	91	M	76	2007	53	Intermediate	3/4	18	0.8	
	92	M	91	2009	45	Severe	5	0	0.0	
	93	F	87	2009	30	Severe	5	0	8.3	
	94	F	92	2009	56	Severe	5	0	0.0	
	95	F	85	2009	31	Severe	5	0	0.0	
	96	M	88	2007	18	Severe	6	0	0.0	
	97	M	65	2001	5	Intermediate	3	12	7.5	
	98	F	84	2008	14	Intermediate	4	18	3.0	
	99	M	80	2007	25	Intermediate	3	30	0.0	
	100	M	83	2003	38	Intermediate	3	0	0.0	
	101	F	80	2009	28	Intermediate	4	0	0.0	
	102	M	74	2007	18	Low	2	0	2.6	
	103	M	79	2005	4	Intermediate	3	11	7.0	
	104	F	70	2006	23	Intermediate	3	0	0.0	
	105	M	83	2006	4	Intermediate	4	7	nd	
	106	M	86	2005	8	Low	2	29	0.0	
	107	F	92	2006	55	Intermediate	3	0	0.0	
	108	M	79	2008	12	Low	1	0	0.0	
	109	M	69	1999	21	Intermediate	3	20	nd	
	110	M	82	2001	55	Low	1/2	25	2.5	
	111	M	90	2004	48	Severe	5	0	nd	
	112	M	80	2004	84	Severe	5	18	nd	
	113	F	88	2004	0	Intermediate	3	20	nd	
	114	F	88	2005	24	Severe	6	0	nd	
										
AD	115	M	80	2009	10	Severe	6	8	15.0	
patients	116	F	84	2009	25	Severe	4	0	5.5	
	117	F	85	2009	79	Severe	6	0	5.0	
	118	F	85	2010	20	Severe	6	0	5.3	
	119	F	88	2008	44	Severe	5	16	3.0	
	120	F	90	2009	74	Severe	5	13	nd	
	121	F	86	2008	14	Severe	6	3	4.5	
	122	F	98	2009	24	Severe	4	15	2.5	
	123	M	88	2009	29	Severe	6	17	1.0	
	124	M	82	2008	70	Severe	4	17	1.3	
	125	F	72	2011	67	Severe	6	0	5.0	
	126	F	103	2009	12	Severe	5	0	nd	
	127	F	84	2010	30	Severe	6	6	1.0	
	128	M	97	2009	18	Severe	5	15	0.3	
	129	F	98	2010	25	Severe	6	0	2.5	
	130	M	88	2009	18	Severe	6	19	1.0	
